# Towards a Functional Approach to the Assessment of Daily Life Physical Activity in Children: Are the PAQ-C and Fitbit Flex-2 Technically Adequate?

**DOI:** 10.3390/ijerph17228503

**Published:** 2020-11-17

**Authors:** Fotini Venetsanou, Kyriaki Emmanouilidou, Konstantinos Soutos, Sofoklis A. Sotiriou, Leire Bastida, Ana Moya, Antonis Kambas

**Affiliations:** 1 School of Physical Education and Sport Science, National and Kapodistrian University of Athens, 17237 Athens, Greece; fvenetsanou@phed.uoa.gr; 2 School of Physical Education and Sport Science, Democritus University of Thrace, 69100 Komotini, Greece; kemmanou@phyed.duth.gr; 3 EllinoGermaniki Agogi, 153 51 Athens, Greece; soutos@ea.gr (K.S.); sotiriou@ea.gr (S.A.S.); 4 TECNALIA, Basque Research and Technology Alliance (BRTA), 48160 Derio, Spain; leire.bastida@tecnalia.com (L.B.); ana.moya@tecnalia.com (A.M.)

**Keywords:** validity, reliability, Actigraph GT3X+, self-report

## Abstract

Considering the need for functional physical activity (PA) measures in PA settings, this study sought to determine the technical adequacy of the Physical Activity Questionnaire for Older Children (PAQ-C) and the Fitbit Flex-2, two instruments with promising features for wide use, using the Actigraph GT3X+ accelerometer as the criterion reference. A total of 218 Greek children (94 boys, 124 girls; mean age = 10.99 ± 1.52 years) volunteered to join in. Participants wore the PA trackers for seven days and completed the PAQ-C. Moreover, a sub-group (*n* = 60) recompleted the PAQ-C after a week. Results revealed acceptable internal consistency and excellent test–retest reliability for the PAQ-C. Regarding concurrent validity, weak to moderate correlations with PA parameters recorded by the GT3X+ were revealed for the total PAQ-C and were excellent for the Flex-2, while a Bland–Altman plot indicated good agreement. Finally, in alignment with relevant literature, significant gender, but no age, differences were found in participants’ PA records in all the tools applied. The above results support the use of the PAQ-C and the Fitbit Flex-2 in children. Considering that they shed light into different parameters of children’s habitual PA, their combined utilisation, providing comprehensive information, is strongly recommended.

## 1. Introduction

The importance of children’s participation in physical activity (PA) not only for obesity reduction [1], but also for the enhancement of several health aspects [2,3,4,5,6], academic achievement [7], and lifelong promotion of PA [6] is well established. Based on the above, the WHO recommends that for positive health outcomes, children and youth should accumulate at least 60 min of moderate-to-vigorous physical activity (MVPA) every day [8].

Nevertheless, today’s children have adopted sedentary behaviours that have resulted in noticeable reduction of PA [9,10,11,12] and increases in childhood obesity to worrying levels [13] that call for efficient policies to protect public health [14]. If such policies are to be designed and implemented, the accurate and functional assessment of PA is of paramount importance, since it allows for a deeper understanding of children’s PA profile and contributes to the evaluation of policies’ and/or interventions’ efficacy [15]. For that purpose, a valid and reliable instrument that can be widely applied in daily life (low cost and easy to use and interpret for children, parents and teachers) is needed.

Several PA assessment tools, both subjective and objective, are available; however, the fact that PA is multifaceted (involving behaviours that relate to transport, physical education, recess, participation in sports/leisure activities, etc.) makes its measurement so complicated that there is no ideal tool perfectly describing all the aspects of PA and well-suited for wide use [16]. Starting with the objective assessment methods, the gold standard for energy expenditure is thought to be the doubly labelled water method, which is, however, expensive and is difficult to apply in daily life [17]. Among the wearable monitoring devices, accelerometers are widely recommended as the most valid and reliable PA measures [18,19], whereas the use of pedometers is supported due to the fact that they are inexpensive and provide information that the general public can understand [20]. Nevertheless, both the above devices are not without shortcomings. To begin with, accelerometers seem unsuitable for PA recording in settings such as schools or sport clubs, due to their cost, which is still high, and the requirements in technical expertise for their use. Moreover, both accelerometers and pedometers present several administration issues (e.g., participants forget or do not want to wear them) that cannot be ignored.

During the last decade, commercially available wearable activity trackers have become very popular. Among the most popular are the Fitbit devices (Fitbit Inc, San Francisco, CA, USA) prevailing in the global wearables market [21]. The FitBit trackers have several features that make their use by children advantageous (inexpensive, in wristband form, can be worn 24 h/day, waterproof). Moreover, the fact that they provide free access to an online database makes them a promising educational means for projects aiming at enhancing children’s motivation to be more active [22]. However, very few studies provide evidence regarding the technical adequacy of these devices’ use in children [23,24,25], restricting a sound conclusion.

Self-report PA assessment tools (e.g., diaries, questionnaires) can provide useful information that can be exploited if children’s PA habits are to be targeted and/or evaluated. Thus, these tools could be valuable alternatives to the objective ones for large-scale use, since they are cost-effective, relatively quick to answer and easy to use for non-specialised staff; thus, they enable recording of PA in large samples in a short period [26,27,28,29]. Nevertheless, they seem to have several weaknesses, such as inaccuracies, over-reporting PA, problematic use in young children [30,31] and insufficient technical adequacy [27,32]. That is why very few self-report measures are recommended as valuable PA tools [27,32,33,34]. Among them is the Physical Activity Questionnaire for Older Children (PAQ-C), a simple seven-day recall questionnaire that aims at providing a global measure of PA during the school year in 8–14-year-old children [35,36]. The PAQ-C has been translated into several languages around the world (e.g., Dutch [37], Chinese [38], Tunisian [16], Japanese [39]), enabling large-scale research into older children’s PA in these countries as well as cross-cultural comparisons.

It appears that both the Fitbit trackers and the PAQ-C have promising features that allow their use in school and physical activity settings, where it is important for PA measures to be accurate as well as functional. However, research evidence regarding their validity and reliability is limited. Furthermore, to our knowledge, there is no published study examining how accurate those two tools are against the same criterion. Based on the above, this study aimed at determining the technical adequacy of the PAQ-C and the Fitbit Flex-2, using the Actigraph GT3X+ accelerometer as the criterion reference.

## 2. Materials and Methods

### 2.1. Participants

An invitation for participation was sent to a convenient sample of parents/legal guardians whose children attended the fourth or seventh grade of two elementary and two secondary schools (Attica region, Greece), informing them in detail about the purpose and the procedures of the study. Two hundred and seventy-two potentially eligible participants responded by returning their signed consent form; however, the data of those who did not meet the criteria of objective PA measurements (*n* = 37) or answered “yes” to the 10th question of the PAQ-C (*n* = 17), which asks the examinee if he/she was sick in the past week, were excluded from the analyses. Finally, 218 children (94 boys, 124 girls; mean age = 10.99 ± 1.52 years) constituted the final sample of the study. Among them, 90 (39 boys, 51 girls) attended the fourth grade (mean age = 9.85 ± 0.28 years) and 128 (55 boys, 73 girls) the seventh grade (mean age = 12.94 ± 0.30 years). No differences in age and body mass index (BMI) scores existed between the final participants and those excluded from the analyses. The present study was part of the research project named “Smart childhood Obesity CARing solution using IoT potential—OCARIoT”, which was approved by the Ethics Committee of the Democritus University of Thrace (approval number 4/33/2018).

### 2.2. Measures

#### 2.2.1. Anthropometry

Standing height and body mass measurements were conducted in a private room at each school, always in the presence of an assistant of the same gender. Children were barefoot and wore light clothing. Standing height (accurate to 0.5 cm) was measured using a stadiometer (Stadiometer 208, Seca, UK) and body mass (accurate to 0.1 kg) using a mechanical scale (Beam Balance 710, Seca, UK). Children’s BMI was calculated with the formula: body mass/height^2^ (kg/m^2^).

#### 2.2.2. Physical Activity

Participants’ PA was recorded with the PAQ-C [36], the Fitbit Flex-2 (Fitbit Inc, San Francisco, CA, USA) and the Actigraph GT3X+ accelerometer (Actigraph, Pensacola, FL). The PAQ-C [36] is a self-administered, 7-day recall instrument with 10 items developed to assess general levels of PA (playing sports, games, doing dance, or any other PA) in children 8–14-years old. The first item contains a list of 22 activities and the examinee is asked to check how many times he/she has done any of them over the last week. The 2nd to 9th items refer to (a) the frequency of children’s participation in physical activities (during physical education lessons, right after school, in the evenings, etc.) and (b) what they did during recess and lunch time at school. Finally, the 10th item is used to identify those who had faced anything that prevented normal PA the week before. For its cultural adaptation, the original version of the PAQ-C [35,36] was translated into Greek by two members of the research team who are native Greek speakers with English fluency. Then, two external bilingual reviewers (native English speakers with Greek fluency) back-translated this Greek version into English, and finally, the original, Greek and back-translated versions of the PAQ-C were checked and discussed in terms of wording and cultural concepts by the research team and external reviewers. That procedure resulted in removing some of the activities presented in the first item that were not familiar to Greeks (i.e., American football, street hockey, field hockey, cross-country, and ice hockey).

The Fitbit Flex-2 (Fitbit Inc., San Francisco, CA, USA) is a tri-axial, wrist-worn accelerometer that includes a 5 LED light monitor to update the progress of the PA, recording data at 100 Hz epochs; it can be worn 24 h a day during all kind of activities and its battery lasts up to 7 days. Moreover, the https://www.fitbit.com platform enables data saving and management in xls file format. According to its manual, the Flex-2 records steps, active minutes, sleep duration, sedentary time and calories. In the present study, only the step counts were used.

The Actigraph GT3X+ accelerometer (Actigraph, Pensacola, FL, USA) is highly recommended as a valid and reliable tool for the evaluation of PA [40,41]. It uses a Micro-Electro-Mechanical System sensor to collect data as digital counts at a predetermined epoch. In this study, a 5-sec epoch for PA recording was set, while the cut-off points proposed by Evenson et al. [18] were used to define light PA (LPA: <2296 counts/min), moderate PA (MPA: <4012 counts/min) and vigorous PA (VPA: ≥4012 counts/min). According to the recommendations of Esliger et al. [42], values >15,000 counts/min should be excluded from the data, implying an instrument malfunction. ActiLife Data Analysis Software version 6.2 was used to store and analyse the accelerometer data.

### 2.3. Procedure

A briefing meeting took place in each school to familiarise parents and children with the use of GT3X+ and Fitbit devices. At the end of that meeting, each parent was provided with a pre-initialised GT3X+ accelerometer attached to a belt and a Fitbit Flex-2. Participants wore the Fitbit on their right wrist and the GT3X+ with the adjustable elastic belt on their hip for seven consecutive days during waking hours. Only data of children who wore the accelerometer and the activity tracker for ≥4 days (ideally one being a weekend day) were included in the analysis. On the day, the devices were returned, the Greek version of PAQ-C was administered to the participants in a quiet room during school time. Their responses concerned PA of the previous week [36]. One week later, the PAQ-C was readministered to a sub-sample of 60 children (24 boys, 36 girls; mean age = 11.35 ± 1.54 years) to check its test–retest reliability. Data were collected from April to June 2019.

### 2.4. Statistical Analyses

Descriptive statistics are presented as means and standard deviations (M ± SD) or relative frequencies. Multivariate analyses of variance were used to examine potential differences between the two grades and genders in PAQ-C total and item scores, MVPA (minutes/day) and steps/day recorded by the GT3X+ and the Flex-2. The internal consistency of the PAQ-C was evaluated computing Cronbach’s alpha coefficient on the total sample as well as on each grade separately, with values ≥0.70 considered acceptable [43]. Furthermore, the intraclass correlation coefficient (ICC) was performed to check its test–retest reliability. For the concurrent validity of both the PAQ-C and the Fitbit Flex-2 against PA parameters provided by GT3X+ (MVPA and steps/day), Spearman’s rank correlation coefficients (for the PAQ-C) and Pearson correlation coefficients (for the Fitbit Flex-2) were calculated, while the Bland–Altman method [44] was utilised to check the agreement between the criterion reference and data obtained by the PAQ-C (total score) and the Flex-2, after transforming them into z scores. Finally, the Spearman’s ρ was used to check the association between the PAQ-C scores and the Fitbit Flex-2 PA records. Correlations <0.29 were considered “weak”, between 0.30 and 0.39 “moderate”, between 0.40 and 0.69 “strong” and those above 0.70 were considered “very strong” [45]. The IBM SPSS 20.0 (Chicago, IL, USA) software package was used to perform data analysis.

## 3. Results

Descriptive characteristics of the participants and their scores in PA measurements according to their grade and gender are reported as means and SDs in Table 1. As far as potential PA differences between age groups and/or genders are concerned, the analyses of variance showed that there were no significant interactions between the two factors or significant differences associated with age. Nevertheless, boys presented statistically significantly higher total PAQ-C scores (F = 12.41, *p* < 0.005), MVPA (F = 91.27, *p* < 0.001), and steps/day (F = 27.09, *p* < 0.001 for the GT3X+; F = 41.04, *p* < 0.001 for the Flex-2, respectively) than girls. Furthermore, a closer look at the PAQ-C individual items revealed significant differences favouring boys in item 3 (F = 38.08, *p* < 0.001), item 5 (F = 15.54, *p* < 0.001) and item 7 (F = 11.18, *p* < 0.005).

As far as the PAQ-C’s internal consistency is concerned, the Cronbach’s α was found to be 0.73 for the total sample, 0.71 for the fourth grade and 0.78 for the seventh grade, all above the 0.70 cut-off. Moreover, the results regarding its test–retest reliability revealed high values of intraclass correlation coefficient (ICC) for the total sample (ICC = 0.974, *p* < 0.001, CI = 0.915–0.969) as well as for the fourth-grade (ICC = 0.964, *p* < 0.001, CI = 0.924–0.983) and the seventh-grade participants (ICC = 0.985, *p* < 0.001, CI = 0.968–0.993).

**Figure 1:**
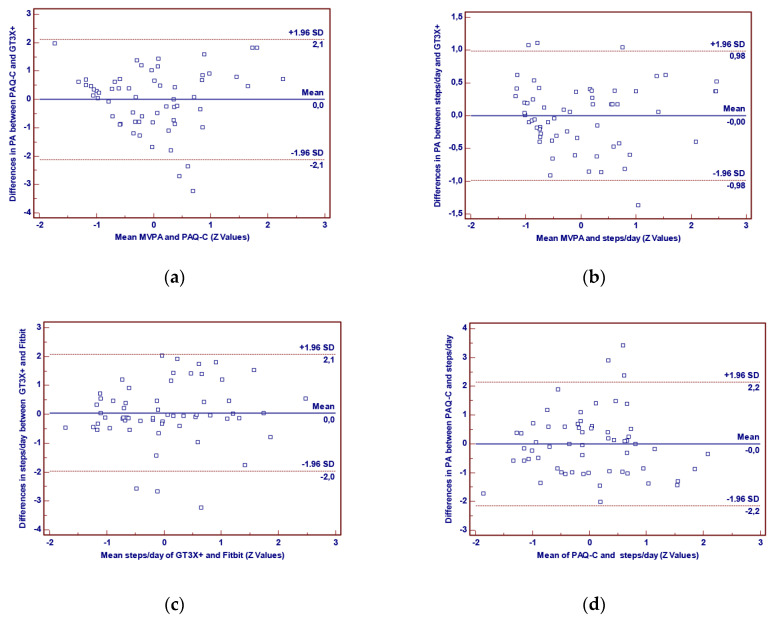
Bland–Altman plots with differences for (**a**) PAQ-C total score and MVPA, (**b**) Flex-2 steps/day and MVPA, (**c**) steps/day of GT3X+ and steps/day of Flex-2, (**d**) PAQ-C total score and Flex-2 steps/day.

In Table 2, the results concerning the concurrent validity of both the PAQ-C and the Fitbit Flex-2, with GT3X+ as the criterion reference, as well as the associations between the PAQ-C and the Fitbit Flex-2, are presented. As can be noticed, Spearman coefficients for the PAQ-C ranged from −0.002 to 0.37, with the total score and some of its items presenting statistically significant correlations with MVPA and step counts recorded by the GT3X+. The associations for the total score were stronger than those for the individual items, achieving moderate correlations with MVPA (ρ = 0.35) and weak correlations with steps/day (ρ = 0.29). Moreover, the Fitbit Flex-2 showed very strong correlations with the GT3X+ data and moderate correlation with the PAQ-C total score, and it also presented statistically significant correlations with five out of nine PAQ-C items. Finally, the Bland–Altman method revealed small differences between all PA measures, indicating a good agreement (Figure 1).

## 4. Discussion

The worrying levels of inactivity and obesity in today’s children, calling for effective policies and interventions towards PA enhancement, have brought the importance of accurate PA assessment to the forefront. For that purpose, tools that are not only accurate but also functional and can be widely applied in daily life are needed. Therefore, this study sought to determine the technical adequacy of the PAQ-C and the Fitbit Flex-2, which both have promising features for wide use (easy to use and interpret, non-invasive, cost- and time-effective). Excellent time stability over a week, sufficient internal consistency and moderate concurrent validity for the PAQ-C, as well as excellent concurrent validity for the Flex-2, using GT3X+ data as the criterion, were revealed, providing support for the use of both instruments in children.

First, Cronbach’s α, performed to check PAQ-C internal consistency, was found to be 0.78 for the 7th grade, a value that was similar to previous PAQ-C adaptations in several countries [37,38,39,46,47]. The estimates for the 4th grade and the total sample were lower; nevertheless, taking into account that a value of Cronbach’s α higher than 0.70 suggests a reliable questionnaire [43], our results suggest satisfactory internal consistency of the PAQ-C in Greek children. As far as its test–retest reliability is concerned, research evidence supports the stability of the PAQ-C over time, since it has been found to present moderate (ICC = 0.73 [26]; 0.75 [48]), good (ICC = 0.82 [38]; 0.83 [39]) and excellent (ICC = 0.90) [49]; 0.96 [46]) test–retest reliability. The variability of the aforesaid ICCs can be attributed to the wide range of the time intervals used for readministering the PAQ-C in the above studies. For example, Benítez-Porres et al. [46] readministered the PAQ-C on the same day six hours apart, Isa et al. [39] reutilised it after two months and Voss et al. [26] after four months. Such a discrepancy in research designs inevitably resulted in different reliability coefficients. In this study, we used a one-week interval (also used in the original PAQ-C reliability study [50]) to avoid both the risk of learned responses (that might have happened in case of retesting few hours apart) and the possibility that children’s PA habits would have changed (in the case of retesting several weeks later). The values of ICC revealed in our study are higher than those of previous ones in which the same time interval was utilised [38,50], and provide strong evidence supporting excellent stability of the PAQ-C in this population when administered after seven days.

Since accelerometers are thought as one of the most accurate methods for PA assessment [18,19], the concurrent validity of both the PAQ-C and the Fitbit Flex-2 were checked using PA parameters provided by the GT3X+ as the criterion reference. Starting with the PAQ-C, according to the Bland–Altman method, its agreement with the GT3X+ was good. Additionally, its total score achieved a moderate correlation with MVPA (ρ = 0.35) and a weak one with steps/day (ρ = 0.29). The above values are higher than those reported by Benítez-Porres et al. [46] (ρ = 0.25 for MVPA and 0.23 for steps/day) and Wang et al. [38] (ρ = 0.33 for MVPA). On this point, it is noteworthy to mention that those two instruments probably cannot have stronger than moderate associations [51], since they seem to measure different things [52]. Accelerometers assess the duration, intensity and frequency of human movement, whereas the PAQ-C was developed to provide a global measure of children’s PA [36]. Additionally, the latter provides information about types of PA that could not be captured by accelerometers, such as cycling and swimming. In our study, 29% of children replied “yes” for cycling and 41% for swimming, two beneficial physical activities that would not have been recorded by the GT3X+ accelerometers. Furthermore, the fact that children’s ability to achieve detailed recall, especially regarding time, is limited [29] has led several authors to conclude that moderate coefficients reflect high validity for the PAQ-C [27]. Based on the above, our findings are encouraging for use of the PAQ-C.

As far as the concurrent validity of the Fitbit Flex-2 with the GT3X+ data is concerned, the Pearson correlation coefficient that was performed showed very strong associations with both MVPA (r = 0.88) and steps/day (r = 0.94) recorded by the Actigraph GT3X+, whereas the Bland–Altman revealed good agreement. Feehan et al. [53], in their recent review, concluded that the Fitbit activity trackers are likely to accurately record adults’ step counts half the time, tending to overestimate steps in free-living conditions and underestimate them in controlled ones. Similarly, Hamari et al. [24], using the Fitbit One in 9–10-year-old children, found that in MVPA it gives higher step counts compared to the Actigraph. Nevertheless, current results provide strong evidence for the validity of the Fitbit Flex-2 and support several other research works that advocate that Fitbit activity trackers may accurately record PA, serving as a viable alternative to the accelerometers both in adults [54,55] and in young children [23].

Regarding participants’ PA levels, their PAQ-C scores were similar to those found in previous studies in China (2.62 ± 0.68) [38], Japan (2.65 ± 0.68) [39] and Brazil (2.7 ± 0.8) [49]. Nevertheless, scores well above 3.00 were reported in recent studies from Spain (3.24 ± 0.64) [46], Turkey (3.16 ± 0.73) [47] and the UK (3.49 ± 0.68) [56], as well as in validation studies conducted in previous decades [35,50]. Still, the present PAQ-C results are in agreement with very recent research evidence, suggesting that children in Greece are not sufficiently physically active [57,58]. Similarly to the picture provided by the PAQ-C, PA recorded by the Flex-2 and the GT3X+ was on average below the recommendations for both MVPA (60 min) [8] and ambulatory activity (13,000–15,000 steps/day for boys and 11,000–12,000 steps/day for girls) [59]. Furthermore, in the present study, both the PAQ-C and the Flex-2 records, in alignment with the GT3X+ data, demonstrated significant gender differences in children’s PA levels, with boys being more active than girls, a finding that is consistent with previous studies in Greece [57,58] and abroad [16,39,60]. Additionally, there were no PA differences between students of 4th and 7th grade in any of the PA tools, confirming that nowadays, PA levels are already starting to decline in late childhood [10].

Based on the above, the findings of this study are supported by the literature and provide evidence advocating for the use of the PAQ-C and Fitbit Flex-2 in children. However, in agreement with several other research works [33,46,51], we strongly recommend their combined use, since it permits obtaining the most comprehensive information, which will result in a better understanding of children’s habitual PA. The PAQ-C, not requiring a large budget and/or specialised personnel, is a feasible measure for large-scale studies. Moreover, the information it gathers about the type of PA can be valuable for personalised PA prescription and effective interventions in school and PA settings. On the other hand, the Fitbit Flex-2 can offer objective PA data that will overcome the potential inaccuracy of children’s self-reports. Given their ease of use, Fitbit Flex-2 devices can have a multifaceted educational role at schools. To start with, they can provide objective information about how physically demanding the activities in which students engage during recess are and/or how active the lesson of physical education is, helping educators to make necessary adjustments to provide their students an active school environment. Furthermore, the free access to an online database that the Fitbit devices offer can be exploited in several projects aiming at enhancing students’ PA.

To our knowledge, this is the first study examining the technical adequacy of both the PAQ-C and the Fitbit Flex-2 in children, delivering an insight on their accuracy in assessing habitual PA. Furthermore, it is the first reported validation study of the Greek version of the PAQ-C. Evaluating the validity of both instruments against the Actigraph GT3X+ accelerometer undoubtedly constitutes a strength of this study. Nevertheless, there are also some limitations that should be taken into account when interpreting its findings. To begin with, our participants were recruited from the 4th and 7th grades of two elementary and two secondary schools, limiting the generalizability of our results. Further research, including children from the whole age range of the PAQ-C, is necessary if comprehensive conclusions are to be drawn.

## 5. Conclusions

The results of this study provide strong evidence for the test–retest reliability and internal consistency of the PAQ-C, as well as the concurrent validity for the PAQ-C and the Fitbit Flex-2, using Actigraph GT3X+ data as the criterion reference, supporting the use of both instruments in Greek students of 4th and 7th grades. Due to their attractive features, the PAQ-C and the Fitbit Flex-2 can serve as a feasible alternative for expensive PA tools, such as accelerometers, especially in large-scale studies or educational projects at schools. Provided that there are sufficient financial resources, the combined use of those tools will contribute to a deeper understanding of children’s habitual PA that may aid its enhancement.

## Figures and Tables

**Table 1:** Descriptive statistics of study participants by age group and gender.

	Fourth Grade (*n* = 90)		Seventh Grade (*n* = 128)	
	Boys (*n* = 39)	Girls (*n* = 51)	Boys (*n* = 55)	Girls (*n* = 73)
Age (years)	9.91 ± 0.28	9.81 ± 0.28	12.86 ± 0.29	12.99 ± 0.31
Height (metres)	1.36 ± 0.05	1.34 ± 0.04	1.58 ± 0.05	1.59 ± 0.06
Weight (kilograms)	30.55 ± 6.10	29.70 ± 5.38	50.02 ± 8.70	46.93 ± 9.68
BMI ^a^ (kg/m^2^)	16.36 ± 2.72	16.46 ± 2.55	19.88 ± 3.00	18.40 ± 3.31
PAQ-C ^b^				
Item 1: Activity checklist	1.55 ± 0.28	1.52 ± 0.27	1.42 ± 0.19	1.37 ± 0.20
Item 2: Physical education	4.56 ± 0.91	4.18 ± 1.21	4.67 ± 0.67	4.71 ± 0.63
Item 3: Recess	4.33 ± 1.03	3.71 ± 1.08	2.94 ± 1.02	2.03 ± 0.50
Item 4: Lunch	1.59 ± 1.04	1.71 ± 0.90	1.47 ± 0.79	1.14 ± 0.41
Item 5: After school (14 to 18 h)	2.74 ± 1.48	2.35 ± 1.28	2.93 ± 1.49	1.96 ± 0.79
Item 6: Afternoon (18 to 22 h)	2.54 ± 1.19	2.33 ± 0.99	3.05 ± 1.09	2.56 ± 1.20
Item 7: Weekend	2.61 ± 1.18	2.25 ± 1.16	2.58 ± 0.87	2.01 ± 0.77
Item 8: Frequency last week	2.18 ± 1.25	2.37 ± 1.14	3.47 ± 0.88	2.64 ± 0.95
Item 9: Week summary	2.22 ± 0.70	2.16 ± 0.84	2.45 ± 0.51	2.22 ± 0.74
Total PAQ-C score	2.70 ± 0.55	2.51 ± 0.53	2.78 ± 0.37	2.35 ± 0.47
MVPA ^c^ (min/day)	42.46 ± 12.46	31.70 ± 9.21	40.33 ± 11.95	33.31 ± 8.41
Steps/day (GT3X+)	12376 ± 3523	9614 ± 2470	12169 ± 3716	9507 ± 2313
Steps/day (Fitbit Flex-2)	12996 ± 3428	9968 ± 2301	12245 ± 3443	8966 ± 2124

^a^ BMI: body mass index; ^b^ PAQ-C: Physical Activity Questionnaire—Children; ^c^ MVPA: moderate to vigorous physical activity.

**Table 2:** Correlations among PA data provided by the PAQ-C, the Fitbit Flex-2 and the GT3X+.

	MVPA ^a^/Day (GT3X+)	Steps/Day	Steps/Day (Flex-2)
Item 1: Activity checklist	0.113	0.132	0.161
Item 2: Physical education	0.241 **	0.140	0.179 *
Item 3: Recess	0.366 ***	0.311 ***	0.372 ***
Item 4: Lunch	−0.002	−0.026	−0.005
Item 5: After school (14 to 18 h)	0.191 *	0.123	0.166 *
Item 6: Afternoon (18 to 22 h)	0.107	0.092	0.091
Item 7: Weekend	0.276 **	0.298 ***	0.267 **
Item 8: Frequency last week	0.160	0.109	0.135
Item 9: Week summary	0.251 **	0.225 *	0.244 **
Total PAQ-C Score	0.354 ***	0.293 ***	0.325 ***
Steps/day (Flex-2)	0.883 **	0.940 **	

^a^ MVPA: moderate to vigorous physical activity, * 0.05, ** 0.005, *** 0.001.
